# Assessment of Long-Term Outcomes of Lower Limb Fractures Managed by Non-orthopaedic Specialist Surgeons in Rural Rwanda: A Pilot Feasibility Study

**DOI:** 10.7759/cureus.81323

**Published:** 2025-03-27

**Authors:** Lotta Velin, Alex Zhuang, Simon P Bigirimana, Andreas Wladis, Heritier Mfura, Jean-Bertrand A Hakizimana, Ellen Barrett, Cornelia Barth, Abebe Bekele, Robert Riviello, Andreas Meunier, Innocent Niyinkunda, Jules Iradakunda, Barnabas T Alayande

**Affiliations:** 1 Centre for Teaching and Research in Disaster Medicine and Traumatology (KMC), Department of Biomedical and Clinical Sciences, Linköping University, Linköping, SWE; 2 Center for Equity in Global Surgery, University of Global Health Equity, Kigali, RWA; 3 Chobanian and Avedisian School of Medicine, Boston University, Boston, USA; 4 Department of Global Health and Population, Harvard School of Public Health, Boston, USA; 5 Faculty of Medicine, Lund University, Lund, SWE; 6 School of Health Professions, Bern University of Applied Sciences, Bern, CHE; 7 Program in Global Surgery and Social Change, Harvard Medical School, Boston, USA; 8 Department of Orthopedics and Department of Biomedical and Clinical Sciences, Linköping University, Linköping, SWE; 9 Department of Surgery, Butaro Level 2 Teaching Hospital, Kigali, RWA

**Keywords:** east africa, functional and clinical outcome, functional disability, health-related quality of life, long-term outcome, low- and middle-income country, lower extremity trauma

## Abstract

Introduction

Sub-Saharan Africa is disproportionately affected by injuries, and short-term morbidity and mortality are high in this context. Lower limb fractures (LLFs) are a common type of injury that can lead to severe disability with extensive negative social and economic consequences. However, little is known about the long-term outcomes of LLFs in sub-Saharan Africa. In this pilot study, we describe the feasibility of assessing epidemiological patterns of LLFs, the state of care, and long-term outcomes in rural Rwanda.

Methods* *

This study was conducted from July to December 2021. It consists of retrospective data collection from hospital charts and a cross-sectional, phone-based follow-up. Functional outcomes were assessed using the World Health Organization Disability Assessment Schedule (WHODAS; World Health Organization, Geneva, Switzerland) 2.0, and health-related quality of life (QoL) was assessed using a 36-Item Short Form Health Survey questionnaire (SF-36).

Results* *

Eighteen patients were included in the study, of which the majority were males (n=11, 61%), young adults (mean age 29, standard deviation (SD) 25), and without any previous comorbidities (n=16, 89%). Most patients had a single fracture, although nearly one-third presented with multi-trauma (n=5, 28%). The most common injury cause was falls (n=10, 56%). Fractures were most common in the femur/hip (n=10, 56%), and most fractures were closed (n=14, 78%) and non-comminuted (n=9, 50%). Disability was described as high, with a total mean WHODAS summary score of 80 (SD 29), whereas health-related QoL varied across the SF-36 domains, with "general health" being the lowest rated domain with a mean score of 30 (SD 25).

Conclusion

This study highlights the challenges of long-term follow-up after LLFs in a rural, low-resource setting but demonstrates that such a study is feasible if planned with contextual considerations. Self-perceived disability is high, and health-related QoL is low at long-term follow-up, which should be viewed in light of the fact that it primarily affects a young, previously healthy population.

## Introduction

In recent years, global injury mortality has declined, yet in low- and middle-income countries (LMICs), injury remains a common cause of death and disability, with sub-Saharan Africa being particularly affected [1]. This has occurred in parallel with reductions in communicable diseases, leading to injury now being the most common cause of death for young people [1]. Although short-term morbidity and mortality from trauma in LMICs have been documented in single-center, multi-center, and global studies [1-4], long-term outcomes of both general and musculoskeletal trauma are unknown [5]. This partly results from challenges in implementing and maintaining trauma registries, including poor data quality, lack of resources, high loss-to-follow-up rates, and lack of standardized protocols for assessment of long-term trauma morbidity and mortality [6]. The long-term consequences of trauma are likely more pronounced in LMICs, as injured patients face various barriers resulting in delays in seeking, reaching, and receiving care [7-9], including frequent shortages of human and material resources [10]. These barriers also impact access to physical and occupational therapy, assistive technology, rehabilitation, timely diagnosis, and treatment of complications [4,8], essential for effective lower limb care. Moreover, low socioeconomic status itself has been associated with adverse outcomes after injury (anxiety, depression, and worse functional outcomes) [11,12], further increasing the vulnerability of patients with injury in low-resource settings. The lower limbs are a commonly injured anatomic region in Rwanda and other sub-Saharan African countries [13,14], and lower extremity injuries can lead to severe disability, with socioeconomic implications at a personal and societal level [15]. This pilot study aimed to describe the feasibility of assessing epidemiological patterns of lower limb fractures (LLFs) in a rural Rwandan hospital, the state of care for such injuries, and the effect of long-term outcomes on functioning and quality of life (QoL) after such fractures.

## Materials and methods

Study design 

This study consisted of a retrospective collection of injury and treatment data from hospital records and a cross-sectional phone-based follow-up with the same patients.

Defining LLFs

LLFs were defined as fractures of the hip, femur, tibia, fibula or any combination of these. We did not include soft-tissue injuries, joint luxation or subluxation. 

Outcome measures**

Functional outcomes were evaluated using the World Health Organization Disability Assessment Schedule (WHODAS; World Health Organization, Geneva, Switzerland) 2.0 (36-item version) to assess physical, social, and mental functioning after injury [6]. The 36-Item Short Form Health Survey questionnaire (SF-36) was used to assess health-related QoL. The survey tools were translated to Kinyarwanda before using. WHODAS 2.0 has been developed and used in a variety of settings [16], including a Rwandan study of functional status at a 28-day follow-up after severe injury [17], where it has demonstrated strong cross-cultural validity and feasibility in assessing post-injury function. It consists of six domains (cognition, mobility, self-care, getting along, life activities or household, and participation in society), each comprising multiple “items.” These are all built on the same question structure for assessing disability in activities, with response scales including “none,” “mild,” “moderate,” “severe,” and “extreme,” numerically represented as one to five to indicate increasing disability. Return to work was used as a proxy measure for economic repercussions. For simplicity, this was incorporated in the WHODAS 2.0 survey by adding two questions about work status before and after injury (Appendix 1). SF-36 consists of eight sub-scales: physical functioning (PF), role physical (RP), bodily pain (BP), general health (GH), vitality (VT), social functioning (SF), role emotional (RE), and mental health (MH) (Appendix 2). SF-36 [18] has also been widely used in injury research and demonstrated feasibility in sub-Saharan African settings [19,20]. *Study Location* The study was conducted at the Butaro Level Two Teaching Hospital (BL2TH), a rural hospital in Rwanda’s mountainous Northern Province. It serves as a 256-bed district hospital for Burera District and surrounding communities. The hospital has two full-time general surgeons but no orthopedic surgeon. BL2TH classifies as a bellwether-capable hospital (with capacity to perform cesarean sections or C-sections, laparotomies, and surgical management of open fractures), but the surgical caseload is heavily skewed toward C-sections and breast oncologic surgeries. As a result, most patients requiring orthopedic surgery or advanced sub-specialty surgical treatment are referred to Ruhengeri (Musanze) Referral Hospital, which is about a two-hour drive away. BL2TH was purposively chosen due to its rural location, the absence of an orthopedic surgeon, and its proximity to the University of Global Health Equity. The location was considered important since previous studies have indicated a large rural-urban gap in access to trauma care [7]. The absence of an orthopedic specialist was important for generalization to other rural district settings where general surgeons provide orthopedic care. *Study Sample* The study included all eligible patients admitted and treated for LLFs as their primary injury at BL2TH between July 1 and December 31, 2021. The exclusion criteria were defined in Table 1.

**Table 1:** Exclusion criteria for study sample selection. LLF: Lower limb fracture.

Exclusion criteria
No available contact information or participant not accessible using provided contact information and/or through a local community health worker.
Hospital admission for LLF not occurring at least 12 months preceding data collection.
Hospital admission occurring following major generalized trauma with LLFs existing only as a secondary diagnosis.
Hospital admission occurring following isolated soft-tissue injuries or joint luxation of the lower extremities.
Patient suffering from known comorbidities that could influence recovery or follow-up (i.e., diabetes, alcohol or substance abuse, psychiatric illness).
Patient does not speak English or Kinyarwanda.
Patient suffering from cognitive impairment affecting their ability to consent.
Patient has been incarcerated in the period between sustained injury and follow-up.
The permanent residence for the patient is outside of Rwandan borders.

No sample size calculation was conducted, as the aim was exploratory rather than hypothesis-testing. *Data Collection* Eligible patients were identified through a retrospective chart review of paper-based records from the hospital archives. The patient identification numbers were verified in the electronic medical system, where additional data were extracted if available. All data were extracted in a pre-set questionnaire (Appendix 3). The patients were contacted at least 12 months after the injury and were invited to participate in a phone-based follow-up interview. If unable to reach the patient after three attempts, the community health worker (CHW) in the patient’s village was contacted to retrieve patient contact information. If still unavailable, the patient was considered lost to follow-up. *Ethical Considerations* This study was approved by the Rwanda National Ethics Committee (No.141RNEC/2023). Codified data were stored securely on Research Electronic Data Capture (REDCap; Vanderbilt University, Nashville, Tennessee, USA) [21]. A patient key containing their respective study codes was stored in a password-protected Excel (Microsoft Corporation, Redmond, Washington, USA) file. For phone-based follow-up data collection, participants were provided with study information in Kinyarwanda, per the Helsinki Declaration and modeled on the University of Global Health Equity information and consent form. Oral consent was obtained before the interview. *Data Analysis* Descriptive statistics (means, standard deviations or SDs, proportions, and frequencies) were used for primary and secondary endpoints (patient demographics, injury characteristics, clinical, functional, and QoL outcomes). For continuous variables, the assumption of normal distribution was tested, and median and interquartile range (IQR) were reported if the assumption failed. For WHODAS, mean polytomous item scores (where the original coding was preserved rather than dichotomized) were calculated [16], as well as overarching mean and total summary domain scores. For SF-36, a mental component summary and a physical component summary can be calculated using z-distributions based on population norms. Since no such norm scores exist in Rwanda, we only present sub-scale scores. Original sub-scale scores were converted to standardized 0-100 scores [22], with means and SDs presented across each sub-scale, where 100 indicates the highest QoL (Appendix 4). No global SF-36 score was calculated, as this is not supported in the scoring, although this is frequently reported in literature [23]. Analysis was conducted using Stata/BE 18.0 (StataCorp. 2023. Stata Statistical Software: Release 18. College Station, TX: StataCorp LLC).

## Results

Demographic characteristics**

**Figure 1:**
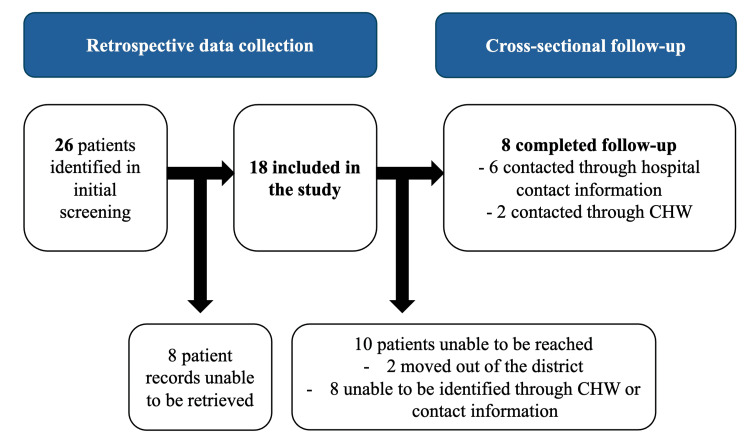
Visualization of the recruitment process with the inclusion and exclusion of study participants. CHW: Community health worker.

In the initial screening of the record book at the surgical and emergency department, 26 patients were identified between July 1 and December 31, 2021. Of these, 18 matching records in the hospital archives were successfully retrieved and included in the study (Figure 1). The majority were male (n=11, 61%), young adults (mean age 29 (SD 25)), and without any previous comorbidities (n=16, 89%) (Table 2). 

**Table 2:** Overview of patient characteristics. BL2TH: Butaro Level 2 Teaching Hospital; IQR: Interquartile range. *Gustilo-Anderson Class III. **38 days after discharge.

		N (%)/Median (IQR)
Age (median)		23 (7-44)
Sex	Female	7 (39)
	Male	11 (61)
Employment status before injury	Farmer	6 (33)
	Other employment	2 (11)
	Not employed (age <15 years)	7 (39)
	Not stated	3 (17)
Highest attained education level	High school	2 (11)
	Primary school	3 (16)
	No schooling	5 (26)
	Not stated	9 (47)
Comorbidities	Cardiovascular	2 (11)
	None documented	16 (89)
Type of trauma	Single fracture and no other injuries	11 (61)
	Multi-trauma with one fracture and other injuries	3 (17)
	Multi-trauma with multiple fractures	2 (11)
	Not stated	3 (17)
Trauma etiology	Fall	10 (56)
	Road traffic accident	2 (11)
	Crush injuries	2 (11)
	Violence/assault	1 (6)
	Not stated	3 (17)
Main diagnosis on admission	Femur/hip fracture	10 (56)
	Tibial fracture	4 (22)
	Pelvic fracture	2 (11)
	Fibular fracture	1 (6)
	Other	1 (6)
Open/closed fracture	Closed fracture	14 (78)
	Open fracture*	1 (6)
	Unknown	3 (17)
Comminuted versus non-comminuted fracture	Comminuted	3 (17)
	Non-comminuted	9 (50)
	Unknown	6 (33)
Waiting time to initial treatment (median number of hours)	-	36 (24-60)
Treatment location/referral	Treated at BL2TH only	5 (28)
	Treated at BL2TH initially, then referred to other hospital for continued care	13 (72)
Treatment at BL2TH	Non-surgical treatment	13 (72)
	Surgical treatment (internal fixation)	1 (6)
	Unknown	4 (22)
Complications	Yes (infection)	2 (11)
	None documented	16 (89)
Length of stay at BL2TH (median number of days)	-	3 (1-11)
Planned follow-up at discharge	Yes	10 (56)
	No	8 (44)
Physiotherapist seen during hospital stay	Yes	1 (6)
	No	17 (94)
Walking aid received at discharge	Wheelchair	1 (6)
	Crutches	2 (11)
	None/unknown	15 (83)
Readmitted	Yes (planned)**	1 (6)
	No	17 (94)

Injury characteristics

Most patients had a single fracture, although nearly one-third presented with multi-trauma (n=5, 28%). The most common injury cause was falls (n=10, 56%). Fractures were most common in the femur/hip (n=10, 56%), and most fractures were closed (n=14, 78%) and non-comminuted (n=9, 50%) (Table 2).

Treatment characteristics

The median time to initial treatment was 36 hours (IQR 24-60), and the majority were transferred to another hospital after initial treatment at BL2TH (n=13, 72%). Median length of stay at BL2TH was three days (IQR 1-11), and approximately half had planned follow-up at discharge (n=10, 56%). Only one patient (6%) had seen a physiotherapist during the stay at BL2TH, and only three received a walking aid at discharge (17%) (Table 2).

Follow-up demographics

Eight participants (44%) were followed up (Table 3), with a mean post-injury interview time being 895 days (SD 71). The follow-up sample was slightly older (median age 37, IQR 11-55) than the total study sample, but the majority were still male. Unlike the total sample, there were no participants with multi-trauma in the follow-up sample. Six were successfully contacted through the contact information in the patient records, and two were followed up through the village CHW. Of those unable to be reached, two were identified by their village CHW as having moved to a different area and, hence, were no longer reachable. The remaining eight were not recognized by their CHW and were deemed as not reachable. None declined to participate.

**Table 3:** Characteristics of follow-up sample. BL2TH: Butaro Level 2 Teaching Hospital; IQR: Interquartile range.

		N (%)/Median (IQR)
Age (median)		37 (11-55)
Sex	Female	3 (38)
	Male	5 (63)
Comorbidities	Cardiovascular	1 (13)
	None documented	7 (88)
Type of trauma	Single fracture and no other injuries	7 (88)
	Not stated	1 (13)
Trauma etiology	Fall	5 (63)
	Road traffic accident	1 (13)
	Crush injuries	1 (13)
	Not stated	1 (13)
Main diagnosis on admission	Femur/hip fracture	6 (75)
	Pelvic fracture	1 (13)
	Fibular fracture	1 (13)
Open/closed fracture	Closed fracture	7 (88)
	Not stated	1 (13)
Comminuted vs non-comminuted fracture	Comminuted	2 (25)
-	Non-comminuted	4 (50)
-	Not stated	2 (25)
Waiting time to initial treatment (median number of hours)	-	48 (24-120)
Treatment location/referral	Treated at BL2TH only	1 (13)
	Treated at BL2TH initially, then referred to other hospital for continued care	7 (88)
Treatment at B2TLH	Non-surgical treatment	6 (75)
	Surgical treatment (internal fixation)	1 (13)
	Not stated	1 (13)
Complications	Yes (infection)	1 (13)
	Not stated	7 (88)
Length of stay at BL2TH (days, median)	-	6 (3-12)
Planned follow-up at discharge	Yes	5 (63)
	No	3 (38)
Physiotherapist seen during hospital stay	Yes	1 (13)
	No	7 (88)
Walking aid received at discharge	Wheelchair	1 (13)
	Crutches	1 (13)
	None/unknown	6 (75)
Readmitted	No	8 (100)

Disability and long-term functional outcomes 

**Figure 2:**
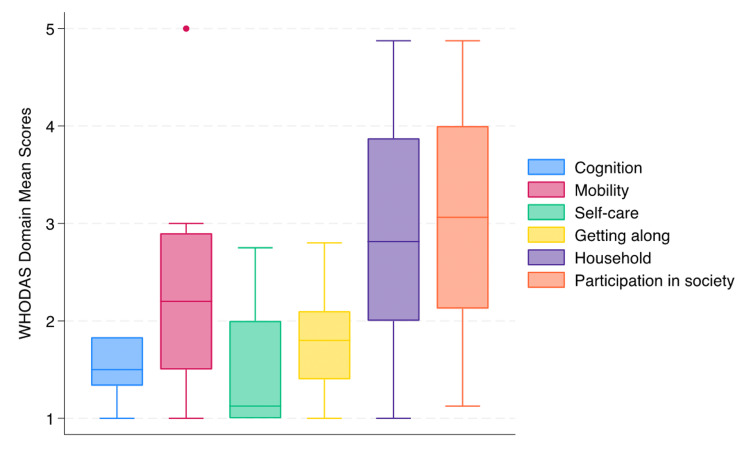
The distribution of WHODAS domain mean scores among the participants, ranging from one to five, representing “no disability” to “extreme disability.” WHODAS: World Health Organization Disability Assessment Schedule.

Of the eight participants with follow-up data, three (38%) were in full-time employment before injury, and five (62%) were in part-time employment. After injury, those who had been in full-time employment remained in full-time employment (n=3), whereas the others transitioned to unemployment (n=5). Two (25%) of those in full-time employment were living independently in the community, whereas the rest (n=6, 75%) resided in a form of assisted living. The total WHODAS mean summary score was 80 (SD 29) on a scale from 36 to 180, corresponding to a scale ranging from “no disability” to “full disability.” The domains with the highest mean summary scores (on a scale from one to five), indicating highest disability, were “participation in the society” (mean 3, SD 0.5), “household” (mean 3, SD 0.2), and “mobility” (mean 2, SD 0.3) (Figure 2; Appendix 5). The items with the highest mean scores were disability relating to sexual activities (item 4.5, mean 4, SD 2), emotional impact (item 6.5, mean 3, SD 1), and impact on the family (item 6.7, mean 3, SD 1).

The domain total summary scores were as follows: cognition (9, SD 2), mobility (23, SD 6), self-care (6, SD 3), getting along (5, SD 2), household (23, SD 11), and participation in society (24, SD 10).

Health-related QoL

The SF36 sub-scales “role limitations due to emotional health” (mean 88, SD 35) and “role limitations due to physical health” (mean 84, SD 35) had the highest health-related QoL scores, and the sub-scales “general health” (mean 30, SD 25) and “vitality” (mean 46, SD 25) had the lowest.

## Discussion

This pilot study describes the feasibility of assessing the epidemiology and long-term functional outcomes of LLFs at a rural Rwandan hospital. The sample in this study indicates a different population demographic compared to the increasingly geriatric population seen in LLF studies in high-income countries [24]. In contrast with other LMIC studies of LLFs [3,25], falls, rather than road traffic accidents (RTAs), were the most common injury cause, and femur/hip fractures were the most common type of fracture, unlike previous injuries indicating the dominance of tibia/patella/fibula/ankle fractures in younger populations [26,27]. Follow-up after a minimum of one year, with a mean follow-up of 2.5 years, indicates high persisting disability, a large impact on functioning in professional capacities, household work, and social functioning, and low health-related QoL. This is especially alarming considering the low sample age. Since the follow-up sample exclusively consisted of patients with single fractures, there may additionally be a survivorship bias [28], excluding the more severely injured multi-trauma patients from the follow-up. Although research is needed at referral hospital levels to assess treatment outcomes, these study results suggest that it may be appropriate to opt for more conservative management in this setting. Surgical treatments such as open reduction and internal fixation (ORIF) are associated with risks and often require timely intervention, experienced staff, availability of adequate materials, and postoperative care, including rehabilitation. In line with this, the surgeons at B2TLH have experience and competency in ORIF procedures but prefer conservative management or refer patients for treatment due to limited time and material availability. Moreover, with load-bearing constructs such as internal fixation, patients are typically prevented from full weight bearing [29] until sufficient healing occurs, which often takes 6-12 weeks. It is uncertain whether compliance with such restrictions is feasible in a low-resource setting with limited access to rehabilitation services. LLFs and their surgical or non-surgical treatment, particularly periarticular knee fractures, are often complicated by joint stiffness. Reduced knee range of motion results in mobility limitations and impacts various gait phases and the ability to manage slopes and sit-to-stand transfers, which increases energy consumption and fatigue [30]. Reduced joint mobility may be a contributing factor to the persisting disability and reduced health-related QoL in our sample, although further studies focusing on rehabilitation are needed. The golden standard for studying functional outcomes is a physiotherapist assessment, including simple measures, feasible and relevant in the local context, such as maximum walking distance or ability to stand or jump on one leg. One tool that may be considered in future studies to assess functional outcomes is the Activity Independence Measure-Trauma, recently validated in humanitarian settings and suitable for patients with limited health literacy [31]. Access to rehabilitation services in Rwanda is severely limited, like many other LMICs [32]. The WHO Rehabilitation 2030 initiative [33] emphasizes the need for increased focus on rehabilitation, including data collection to understand service gaps and guide solutions. At B2TLH, there is one part-time physiotherapist but no standardized rehabilitation plan for injured patients, and only one patient in this sample saw a physiotherapist, indicating room for quality improvement. Previous studies indicated that even when rehabilitation services were offered, prohibitive costs and limited access to transportation made access to such services difficult for rural patients [34]. Most patients had planned follow-ups with a general practitioner in the outpatient department, who, in case of need, could refer them to higher levels of care. Globally, insufficient human resources limit rehabilitation care, prompting WHO to recommend integrating rehabilitative services into CHW systems. Although CHWs are integral to the Rwandan health system [35], their involvement in rehabilitation is yet to be implemented [32]. The major limitation of this study is the small sample size and the loss of follow-up. Several participants lacked phones and therefore provided the contact information of their partners or neighbours, complicating follow-up. The small sample is also likely a result of circumstances specific to Rwanda, such as the relatively short physical distances, which may facilitate the bypassing of district hospitals, with trauma patients often directly going to secondary hospitals with surgical capacities. Yet, the sample is comparable to a 2013 study of non-obstetric surgical patients in three Rwandan district hospitals, including B2TLH [36]. In this study, 1024 trauma patients sought care across the three hospitals over one year, with B2TLH receiving the lowest number of cases. Among these, only 41 patients (4.1%) received surgical management and were discharged from the district hospital, whereas the majority (n=573, 55.9%) received non-operative care before discharge. The low proportion of RTA patients is likely multifactorial and contrasts previous studies from urban Kigali [17]. Firstly, B2TLH is rurally located, far from major highways, with limited road access and significantly fewer motor vehicles. Most fractures result from falls from heights in this mountainous context, and such accidents are relatively few and far between. Secondly, when RTAs occur, police and/or emergency medical services (EMS) often arrive at the scene and take those requiring healthcare directly to a facility with trauma capacity (i.e., bypassing Butaro). Similarly, the proportion of open fractures may be underrepresented, assuming that severe injuries are more likely to be directly transported to other facilities. Although a large knowledge gap remains regarding injury outcomes in rural settings, future studies should consider that rural patients may bypass lower levels of care (such as health centers and district hospitals) and should therefore include secondary hospitals as study sites. The small sample further spurs questions regarding the representativeness of the study location and the sample, and the generalizability of the findings to other LMICs. Firstly, it is important to note that there is no one “typical” LMIC setting, as this umbrella term encompasses significant heterogeneity. Moreover, Rwanda’s pre-hospital EMS are considered well-developed [7], in contrast to findings from a 2013-2014 survey study that showed the absence of pre-hospital EMS in 33 (61%) of 49 African countries [37]. Previous regional studies have also reported a significant skew of EMS toward urban areas, with only 8.6% of the populations living within EMS coverage. In contrast, Rwanda has nationwide EMS coverage [38], further limiting the generalizability of the Rwandan context to other LMICs. Yet, we believe that certain elements of this study context, such as underdeveloped infrastructure, human resource limitations, and financial shortages, are common in some other LMICs. This suggests that the findings may imply some generalizability, and the lessons learned could be used and contextually adapted in such settings [14]. Another study limitation is that the survey tools have not yet been officially translated to Kinyarwanda or validated in Rwanda. We experienced some difficulties in doing the interviews; however, we perceived that the participants easily understood the questions. One survey-related challenge was that, despite stating that the study did not involve financial compensation, some participants insisted on receiving compensation, and there may be a responder bias in participants having exaggerated their long-term disability to motivate such compensation. Although challenging, we believe that important measures were taken to reduce the effects of responder bias in patient-reported outcome measures such as using validated survey tools with neutrally framed questions and having local Kinyarwanda native speakers, who did not have a healthcare-provider relationship with the participants, conduct interviews. Despite the small sample, this study provides value due to the novel context and focuses on previously unexplored functional outcomes. This pilot study indicates many possible directions for future research and considerations that may be useful when planning such studies. Over half of the patients in this cohort suffered fractures due to falls, with femur/hip fractures being the most common. This raises questions about why and how such severe injuries occur, especially in a young population. Further investigations may highlight possible community or public health interventions to reduce these injuries. Another issue that must be addressed is the median wait time of 1.5 days (36 hours) before initial treatment. One previous qualitative study identified facility overload, lack of trained personnel, medication shortages, and hospital workflow as significant contributors to care delays at Rwandan hospitals [34]. Root cause analyses and process mapping can help identify areas for improvement, as the contributing factors are likely systemic and multifactorial. Future research involving prospective cohorts with a significantly larger sample size will be needed to assess the long-term disability outcomes of trauma patients in rural settings. Randomized controlled trials that address confounders will help provide higher quality evidence.

## Conclusions

This study highlights the challenges of long-term follow-up after LLFs in a rural, low-resource setting but demonstrates that such a study is feasible if planned with contextual considerations. The demographic characteristics of patients with LLFs in rural Rwanda appear to be in stark contrast to the increasingly geriatric population in high-income countries and merit further research to guide contextually relevant treatment guidelines. Self-perceived disability is high, and health-related QoL is low at long-term follow-up, which should be considered in the context of this condition primarily affecting a young, previously healthy population.

## Appendices

Appendix 1. WHODAS 2.l0 36-item survey 

 36-item version

This questionnaire asks about difficulties due to health conditions. Health conditions include diseases or illnesses, other health problems that may be short or long-lasting, injuries, mental or emotional problems, and problems with alcohol or drugs. Think back over the past 30 days and answer these questions, thinking about how much difficulty you had doing the following activities. For each question, please circle only one response.

**Table 4:** World Health Organization Disability Assessment Survey (WHODAS) 2.l0 36-item survey.

Variable	Answer options
Name	-
Date of injury	-
Date of interview	-
Employment status before injury	Full-time employment/part-time employment/unemployed/unable to work
Current employment status	Full-time employment/part-time employment/unemployed/unable to work
Living situation	Independently in the community/assisted living/hospitalized
Cognition - in the past 30 days, how much difficulty did you have in: Concentrating on doing something for ten minutes? Remembering to do important things? Analyzing and finding solutions to problems in day-to-day life? Learning a new task, for example, learning how to get to a new place? Generally understanding what people say? Starting and maintaining a conversation?	None/mild/moderate/severe/extreme/cannot do/unapplicable
Mobility - In the past 30 days, how much difficulty did you have in: Standing for long periods such as 30 minutes? Standing up from sitting down? Moving around inside your home? Getting out of your home? Walking a long distance such as a kilometer (or equivalent)?	None/mild/moderate/severe/extreme/cannot do/unapplicable
Self-care - In the past 30 days, how much difficulty did you have in: Washing your whole body? Getting dressed? Eating? Staying by yourself for a few days?	None/mild/moderate/severe/extreme/cannot do/unapplicable
Getting along - In the past 30 days, how much difficulty did you have in: Dealing with people you do not know? Maintaining a friendship? Getting along with people who are close to you? Making new friends?	None/mild/moderate/severe/extreme/cannot do/unapplicable
Life activities - In the past 30 days, how much difficulty did you have in: Taking care of your household responsibilities? Doing most important household tasks well? Getting all the household work done that you needed to do? Getting your household work done as quickly as needed?	None/mild/moderate/severe/extreme/cannot do/unapplicable
If you work (paid, non-paid, self-employed) or go to school, complete questions below. Otherwise, skip these. Because of your health condition, in the past 30 days, how much difficulty did you have in: Your day-to-day work/school? Doing your most important work/school tasks well? Getting all the work done that you need to do? Getting your work done as quickly as needed?	None/mild/moderate/severe/extreme/cannot do/unapplicable
Participation - In the past 30 days: How much of a problem did you have in joining in community activities (for example, festivities, religious or other activities) in the same way as anyone else can? How much of a problem did you have because of barriers or hindrances in the world around you? How much of a problem did you have living with dignity because of the attitudes and actions of others? How much time did you spend on your health condition, or its consequences? How much have you been emotionally affected by your health condition? How much has your health been a drain on the financial resources of you or your family? How much of a problem did your family have because of your health problems? How much of a problem did you have in doing things by yourself for relaxation or pleasure?	None/mild/moderate/severe/extreme/cannot do/unapplicable
Effect of difficulties - Overall, in the past 30 days, how many days were these difficulties present? Record number of days: In the past 30 days, for how many days were you totally unable to carry out your usual activities or work because of any health condition? Record number of days: In the past 30 days, not counting the days that you were totally unable, for how many days did you cut back or reduce your usual activities or work because of any health condition?	None/mild/moderate/severe/extreme/cannot do/unapplicable

Appendix 2. SF-36 survey 

**Table 5:** SF-36 survey.

Variable	Answer option
Study ID	
In general would you say your health is	Excellent/very good/good/fair/poor
Compared to before the injury, how would you rate your health in general now?	Much better now than before the injury/somewhat better now than before the injury/about the same/somewhat worse now than before the injury/much worse now than before the injury
General health: How true or false is each of the following statements for you? I seem to get sick a little easier than other people. I am as healthy as anybody I know. My health is excellent. I expect my health to get worse.	Definitely true/mostly true/don't know/mostly false/definitely false
The following are about activities you might do during a typical day. Does your health now limit you in these activities? If so, how much? Vigorous activities, such as running, lifting heavy objects, participating in strenuous sports. Moderate activities, such as moving a table, pushing a vacuum cleaner, bowling, or playing golf. Lifting or carrying groceries. Climbing several flights of stairs. Climbing one flight of stairs. Bending, kneeling, or stooping. Walking more than a mile (approx. 1600 m). Walking several blocks. Walking one block. Bathing or dressing yourself.	Yes, limited a lot/yes, limited a little/no, not limited at all
During the past 4 weeks, have you had any of the following problems with your work or other regular daily activities as a result of your physical health? Cut down the amount of time you spent on work or other activities. Accomplished less than you would like. Were limited in the kind of work or other activities. Had difficulty performing the work or other activities (for example, it took extra effort).	Yes/no
During the past 4 weeks, have you had any of the following problems with your work or other regular daily activities as a result of any emotional problems (such as feeling depressed or anxious)? Cut down the amount of time you spent on work or other activities. Accomplished less than you would like. Didn't do work or other activities as carefully as usual.	Yes/no
How much bodily pain have you had during the past 4 weeks?	Very severe/severe/moderate/mild/very mild/none
During the past 4 weeks, how much did pain interfere with your normal work (including both work outside the home and housework)?	Extremely/quite a bit moderately/a little bit/not at all
These questions are about how you feel and how things have been with you during the last 4 weeks. For each question, please give the answer that comes closest to the way you have been feeling. Did you feel full of life? Have you been a very nervous person? Have you felt so down in the dumps that nothing could cheer you up? Have you felt calm and peaceful? Did you have a lot of energy? Have you felt downhearted and blue? Did you feel worn out? Have you been a happy person? Did you feel tired?	All of the time/most of the time/a good bit of the time/some of the time/a little bit of the time/none of the time
During the past 4 weeks, how much of the time has your physical health or emotional problems interfered with your social activities (like visiting with friends, relatives, etc.)?	Very severe/severe/moderately/slightly/none at all

Appendix 3. Demographic, injury, and treatment characteristic survey 

**Table 6:** Demographic, injury, and treatment characteristic survey.

Variable	Answer option
Study ID	
Demographic variables	
Name	
Age	
Sex	Male/female/other
Date of hospital admission	YYYYMMDD
Marital status	Married/divorced/widow(er)/single/other
Employment status	Full-time employment/part-time employment/unemployed/unable to work/other
Employment type	Farmer/factory worker/service industry worker/businessman/business owner/other (if other, please specify)
Highest attained education level	Higher education (after completed high school)/high school or less/primary school or less/no schooling/other
Income level	Ubudehe A/Ubudehe B/Ubudehe C/Ubudehe D/Ubudehe E/other
Insurance status	Mutuelle/other insurance/no insurance/unknown
District of residence	
Sector of residence	
Village of residence	
Number of adults (18+) in household	
Number of children (<18) in household	
Smoking status	Never smoked/former smoker/current smoker/other
Known comorbidities (select all applicable answers)	Cancer/cardiovascular disease/lung disease/HIV/AIDS/diabetes/psychiatric illness/alcohol abuse/substance abuse/other (If other please specify)/none of the above
Acute injury assessment	
Trauma severity	Single fracture and no other injuries/multiple fractures and no other injuries/multi-trauma with one fracture and other injuries (not fractures)/multi-trauma with multiple fractures and other injuries (not fractures)
Other traumatic injury (select all applicable answers)	No other injuries/abdominal/neurosurgical/other (If other, please specify)
Main diagnosis on admission	Pelvic fracture/femur/hip fracture/patellar fracture/tibial fracture/fibular fracture/major generalized trauma/other (If other, please specify)
Side of injury	Right/left/bilateral/unknown
Type of fracture	Comminuted/non-comminuted/unknown
Open fracture	Yes/no/unknown
If open fracture, Gustilo-Anderson classification	1/2/3 (sub-type 3a, 3b or 3c)/unclassified
Blood pressure on admission	Systolic/diastolic mmHg
Pulse on admission	Heart rate/minute
Respiratory rate on admission	Rate/minute
Cause of injury	Road traffic accident/fall injury/blast injury/crush injury/gunshot injury/other
If road traffic accident, type of accident (select all involved)	Pedestrian/motorcycled river or passenger/4-wheel vehicle driver or passenger/other (if other, please specify)
Estimated hours from injury to initial treatment	
Anti-tetanus prophylaxis given	Yes/no/unknown
Antibiotic prophylaxis given	Yes/no/unknown
Received treatment	Surgical treatment other than amputation/amputation/non-surgical treatment/no treatment/other
If surgical treatment, what type (select all applicable)	External fixation/internal fixation/osteosynthesis/traction + cast/closed reduction + cast treatment/other (if other, please specify)
If amputation, specify indication	Vital indication/ischemia/major disability/infection/complicated fracture/other (If other, please specify)
If amputation, specify type	Transfemoral/transtibial/foot amputation/other
If amputation, specify wound closure	Primary closure/delayed primary closure/other
If amputation, specify neurovascular, bone, joint, and soft tissue condition	(free text)
If surgical treatment (amputation or otherwise), specify post-operative treatment received (select all applicable answers)	Wound care/antibiotics/other/none (If other, please specify)
If surgical treatment (amputation or other), subsequent operations conducted	Yes/no
Physiotherapist consultation received	Yes/no
Physiotherapist treatment received	Yes/no (If yes, please specify)
Walking/transport aid received for discharge (select all applicable answers)	Wheelchair/crutches/prosthetic/other/none (If other, please specify)
Complications of injury	Yes/no
Type of complication	Infection/compartment syndrome/post-operative general complications/other (If other, please specify)
Number of days in hospital	
Antibiotic treatment received at discharge	Yes/no
Follow-up appointment	Yes/no (if yes, indication for follow-up)
Readmission to hospital	Yes/no
If readmission, type of readmission	Emergency/planned
If yes, time from discharge to readmission (days)	
If yes, cause of readmission	Surgical/medical/wound related/other (If other, please specify)
If yes, type of complication causing readmission	Fixation failure/pain/deep vein thrombosis/surgical site infection/non-infectious wound problem
Walking/transport aid received for discharge following readmission (select all applicable answers)	Wheelchair/crutches/prosthetic/other/none (If other, please specify)

Appendix 4. SF-36 item list with the original scale format and the converted scale format 

**Table 7:** SF-36 item list with the original scale format and the converted scale format. *This question was originally worded “compared to one year ago, how would you rate your health in general now”, but was reworded for the purposes of our study. **In the original SF-36 Survey, this domain consists of two questions which were merged in this survey to shorten the interviews.

	Original scale format	Converted scale format
Domain 1: Physical functioning (PF)	-	-
PF1: Vigorous activities, such as running, lifting heavy objects	1-3	100, 50, 0
PF2: Moderate activities, such as moving a table, pushing a vacuum cleaner	1-3	100, 50, 0
PF3: Lifting or carrying groceries	1-3	100, 50, 0
PF4: Climbing several flights of stairs	1-3	100, 50, 0
PF5: Climbing one flight of stairs	1-3	100, 50, 0
PF6: Bending, kneeling, stooping	1-3	100, 50, 0
PF7: Walking more than a mile	1-3	100, 50, 0
PF8: Walking several blocks	1-3	100, 50, 0
PF9: Walking one block	1-3	100, 50, 0
PF10: Bathing or dressing yourself	1-3	100, 50, 0
Domain 2: Role limitations due to physical health (RP)	-	-
RP1: Cut down the amount of time you spent on work or other activities.	1-2	0, 100
RP2: Accomplished less than you would like.	1-2	0, 100
RP3: Were limited in the kind of work or other activities.	1-2	0, 100
RP4: Had difficulty performing the work or other activities	1-2	0, 100
Domain 3: Bodily pain (BP)	-	-
BP1: How much bodily pain have you had during the past 4 weeks?	1-6	100, 80, 60, 40, 20, 0
BP2: During the past 4 weeks, how much did pain interfere with your normal work (including both work outside the home and housework)?	1-5	100, 75, 50, 25, 0
Domain 4: General health (GH)	-	-
GH1: General assessment of health	1-5	100, 75, 50, 25, 0
GH2: Assessment of health compared to pre-injury*	1-5	100, 75, 50, 25, 0
GH3: I seem to get sick a little easier than other people	1-5	0, 25, 50, 75, 100
GH4: I am as healthy as anybody I know	1-5	100, 75, 50, 25, 0
GH5: My health is excellent	1-5	100, 75, 50, 25, 0
GH6: I expect my health to get worse	1-5	0, 25, 50, 75, 100
Domain 5: Vitality (VT)	-	-
VT1: Did you feel full of life?	1-6	100, 80, 60, 40, 20, 0
VT2: Did you have a lot of energy?	1-6	100, 80, 60, 40, 20, 0
VT3: Did you feel tired?	1-6	0, 20, 40, 60, 80, 100
VT4: Did you feel worn out?	1-6	0, 20, 40, 60, 80, 100
Domain 6: Social functioning** (SF)	-	-
SF1: During the past 4 weeks, how much of the time has your physical health or emotional problems interfered with your social activities (like visiting with friends, relatives, etc.)?	1-5	0, 25, 50, 75, 100
SF2: Have emotional problems interfered with your normal social activities with family, friends, neighbors or groups?	1-5	0, 25, 50, 75, 100
Domain 7: Role limitations due to emotional health (RE)	-	-
RE1: Cut down the amount of time you spent on work or other activities.	1-2	0, 100
RE2: Accomplished less than you would like.	1-2	0, 100
RE3: Didn’t do work or other activities as carefully as usual.	1-2	0, 100
Domain 8: Mental health (MH)	-	-
MH1: Have you been a very nervous person?	1-6	0, 20, 40, 60, 80, 100
MH2: Have you felt so down in the dumps that nothing could cheer you up?	1-6	0, 20, 40, 60, 80, 100
MH3: Have you felt calm and peaceful?	1-6	100, 80, 60, 40, 20, 0
MH4: Have you been a happy person?	1-6	100, 80, 60, 40, 20, 0
MH5: Have you felt downhearted and blue?	1-6	0, 20, 40, 60, 80, 100

Appendix 5. WHODAS 2.0 domain and item summary scores (mean, standard deviation)

**Table 8:** WHODAS 2.0 domain and item summary scores (mean, standard deviation).

	Mean Score	Standard Deviation
Domain 1: Cognition	1.52	0.419
1.1 Concentrating on doing something for ten minutes?	1.88	0.64
1.2 Remembering to do important things?	1.38	0.52
1.3 Analyzing and finding solutions to problems in day-to-day life?	1.88	0.64
1.4 Learning a new task, for example, learning how to get to a new place?	2.00	1.30
1.5 Generally understanding what people say?	1.00	00
1.6 Starting and maintaining a conversation?	1.00	0.00
Domain 2: Mobility	2.40	0.26
2.1 Standing for long periods such as 30 minutes?	2.38	1.30
2.2 Standing up from sitting down?	2.50	1.41
2.3 Moving around inside your home?	2.13	1.25
2.4 Getting out of your home?	2.13	1.25
2.5 Walking a long distance such as a kilometer (or equivalent)?	2.88	1.46
Domain 3: Self-care	1.51	0.23
3.1 Washing your whole body?	1.63	0.92
3.2 Getting dressed?	1.63	0.92
3.3 Eating?	1.13	0.35
3.4 Staying by yourself for a few days?	1.63	0.92
Domain 4: Getting along	1.60	1.10
4.1 Dealing with people you do not know?	1.25	0.46
4.2 Maintaining a friendship?	1.50	0.76
4.3 Getting along with people who are close to you?	1.25	0.46
4.4 Making new friends?	1.38	0.52
4.5 Sexual activities?	3.63	2.20
Domain 5: Household	2.96	0.24
5.1 Taking care of your household responsibilities?	2.63	1.30
5.2 Doing most important household tasks well?	2.63	1.19
5.3 Getting all the household work done that you needed to do?	2.63	1.41
5.4 Getting your household work done as quickly as needed?	3.13	1.36
5.5 Your day-to-day work/school?	3.13	1.73
5.6 Doing your most important work/school tasks well?	3.00	1.69
5.7 Getting all the work done that you need to do?	3.13	1.36
5.8 Getting your work done as quickly as needed?	3.00	1.20
Domain 6: Participation in society	3.10	0.45
6.1 How much of a problem did you have in joining in community activities (for example, festivities, religious or other activities) in the same way as anyone else can?	2.63	1.41
6.2 How much of a problem did you have because of barriers or hindrances around you?	2.75	1.49
6.3 How much of a problem did you have living with dignity because of the attitudes and actions of others?	2.89	1.46
6.4 How much time did you spend on your health condition or its consequences?	2.89	1.13
6.5 How much have you been emotionally affected by your health condition?	3.38	1.10
6.6 How much has your health been a drain on the financial resources of you or your family?	3.25	1.58
6.7 How much of a problem did your family have because of your health problems?	3.38	1.18
6.8 How much of a problem did you have in doing things by yourself for relaxation or pleasure?	3.25	1.59
